# Effect of a Stewardship Intervention on Adherence to Uncomplicated Cystitis and Pyelonephritis Guidelines in an Emergency Department Setting

**DOI:** 10.1371/journal.pone.0087899

**Published:** 2014-02-03

**Authors:** Michelle T. Hecker, Clinton J. Fox, Andrea H. Son, Rita K. Cydulka, Jonathan E. Siff, Charles L. Emerman, Ajay K. Sethi, Christine P. Muganda, Curtis J. Donskey

**Affiliations:** 1 Department of Medicine, Division of Infectious Diseases, MetroHealth Medical Center, Case Western Reserve University, Cleveland, Ohio, United States of America; 2 School of Medicine, Case Western Reserve University, Cleveland, Ohio, United States of America; 3 Department of Pharmacy, MetroHealth Medical Center, Cleveland, Ohio, United States of America; 4 Department of Emergency Medicine, MetroHealth Medical Center, Case Western Reserve University, Cleveland, Ohio, United States of America; 5 Department of Population Health Sciences, University of Wisconsin, Madison, Wisconsin, United States of America; 6 Geriatric Research, Education and Clinical Center, Louis Stokes Veterans Affairs Medical Center, Cleveland, Ohio, United States of America; Northwestern University, United States of America

## Abstract

**Objective:**

To evaluate adherence to uncomplicated urinary tract infections (UTI) guidelines and UTI diagnostic accuracy in an emergency department (ED) setting before and after implementation of an antimicrobial stewardship intervention.

**Methods:**

The intervention included implementation of an electronic UTI order set followed by a 2 month period of audit and feedback. For women age 18 – 65 with a UTI diagnosis seen in the ED with no structural or functional abnormalities of the urinary system, we evaluated adherence to guidelines, antimicrobial use, and diagnostic accuracy at baseline, after implementation of the order set (period 1), and after audit and feedback (period 2).

**Results:**

Adherence to UTI guidelines increased from 44% (baseline) to 68% (period 1) to 82% (period 2) (*P*≤.015 for each successive period). Prescription of fluoroquinolones for uncomplicated cystitis decreased from 44% (baseline) to 14% (period 1) to 13% (period 2) (*P*<.001 and *P* = .7 for each successive period). Unnecessary antibiotic days for the 200 patients evaluated in each period decreased from 250 days to 119 days to 52 days (*P*<.001 for each successive period). For 40% to 42% of cases diagnosed as UTI by clinicians, the diagnosis was deemed unlikely or rejected with no difference between the baseline and intervention periods.

**Conclusions:**

A stewardship intervention including an electronic order set and audit and feedback was associated with increased adherence to uncomplicated UTI guidelines and reductions in unnecessary antibiotic therapy and fluoroquinolone therapy for cystitis. Many diagnoses were rejected or deemed unlikely, suggesting a need for studies to improve diagnostic accuracy for UTI.

## Introduction

Acute uncomplicated cystitis and pyelonephritis are common indications for prescription of antimicrobials in healthy, non-pregnant women [1]. Guidelines for the treatment of these conditions were first published by the Infectious Diseases Society of America (IDSA) in 1999[2]. Several studies subsequently documented that prescribing practices for uncomplicated urinary tract infection (UTI) varied widely and adherence to these and other UTI guidelines was often low [3]–[13]. The updated IDSA and European Society for Microbiology and Infectious Diseases guidelines published in 2011 emphasize the importance of local susceptibility data and the ecological adverse effects of antimicrobial therapy, including antimicrobial resistance and *Clostridium difficile* infection [14]. Ecological effects merit particular consideration in treatment of cystitis because there is minimal risk of progression to severe illness and infection may resolve in 25% to 42% of women who are not treated or who are treated with a drug without in vitro activity against the uropathogen [14]–[16]. As in the 1999 guidelines, the updated guidelines continue to recommend that in areas in which trimethoprim-sulfamethoxazole (TMP-SMX) resistance rates for uropathogens causing acute uncomplicated cystitis do not exceed 20%, TMP-SMX for 3 days is recommended as a first line option. Fluoroquinolones for 3 days are recommended as a second line option for treatment of cystitis due to their adverse ecological effects [17]. The updated guidelines are more specific in recommending that nitrofurantoin for 5 days or a single dose of fosfomycin should also be considered as first line options for treatment of cystitis. Fluoroquinolones remain a first line option for pyelonephritis; however the updated guidelines more specifically recommend a 7 day fluoroquinolone regimen as opposed to a 7–14 day regimen.

In a recent commentary, May et al. [18] issued a call to action for research to address the large gap in the literature evaluating ED-based antimicrobial stewardship strategies. Uncomplicated UTIs are often diagnosed and treated in the emergency department (ED), [19] however, we are unaware of data on adherence to UTI guidelines or on the effect of antimicrobial stewardship interventions for UTI in this setting. Moreover, some studies have provided evidence that the accuracy of UTI diagnoses may be suboptimal in the ED [20]–[23]. For example, Shapiro et al. [20] found that 43% of patients with traditional UTI symptoms had negative urine cultures. Huppert et al. [22] demonstrated that 53% of adolescent women with a final diagnosis of a UTI had a sexually transmitted infection.

Here, we assessed adherence to guidelines for management of uncomplicated UTI in an urban ED before and after initiation of a stewardship intervention that included implementation of an electronic order set followed by audit and feedback. We also assessed UTI diagnostic accuracy before and after the intervention. We hypothesized that increased adherence to the guidelines would reduce unnecessary days of antibiotic therapy for UTI and reduce the use of fluoroquinolones for uncomplicated cystitis without increasing adverse events or the frequency of treatment failure. We also hypothesized that UTI diagnostic accuracy would improve after audit and feedback.

## Methods

### Study design and setting

We conducted a before and after study between 2010 and 2012 at MetroHealth Medical Center. MetroHealth Medical Center is an academic urban level 1 trauma center averaging 90,000–100,000 ED visits annually of which approximately 200–300 visits per month include any UTI- associated diagnosis, including both uncomplicated and complicated UTIs. The ED at our institution began using an electronic medical record (EMR), EPIC® system, in 1999.

### Ethical approval

The study was conducted in accordance with the Declaration of Helsinki and was approved by the MetroHealth Medical Center’s Institutional Review Board. The Institutional Review Board provided a waiver of the requirement for informed consent as the intervention was deemed to be a quality improvement initiative that involved minimal risk to subjects and no procedures for which written consent would normally be required outside of the research context, the study would not have been feasible without the waiver of consent, and subject medical record review was conducted retrospectively.

### Selection of study patient visits for retrospective review

Potential study patient visits were identified using the following *International Classification of Diseases, Ninth Revision, Clinical Modification* (ICD-9-CM) codes for UTIs: 599.0 for UTI, 595.9 for cystitis, and 590.10 for pyelonephritis. Patient visits for women between the ages of 18 and 65 were randomly selected for review using computer-generated randomization (www.random.org) during twelve specified months over a 3 year time period (2010 – 2012). Patient visits for women with any of the following characteristics were excluded from the study: pregnancy, known significant structural or functional urological abnormalities, urinary tract instrumentation within the preceding 7 days, residency in a skilled nursing facility, inability to give a history, or currently on suppressive antibiotic therapy to prevent UTIs. Patients with diabetes were included unless they had diabetes that was poorly controlled, defined for the purposes of this study as having a hemoglobin A1C greater than 8. Patients with a history of kidney stones were included unless stones were documented to be present at the time of the ED visit. Two hundred patient visits (50 during each of 4 specified months February, March, November, and December 2010) before any intervention (baseline), 200 patient visits (50 during each of 4 specified months February, March, September, October 2011) after the electronic order set intervention (period 1), and 200 patient visits (50 during each of 4 specified months February, March, September, October 2012) after the audit and feedback intervention (period 2) meeting inclusion criteria were included in the study.

### Data collection

Demographic and clinical data were abstracted from the electronic medical record by three of the authors (CJF, MTH, and AHS) using a standardized data collection form with detailed instructions on how each variable was defined. Each chart was reviewed at least twice for accuracy of data collection. Provider determined UTI diagnoses were based on the ICD-9-CM codes (both primary and secondary) attached to the visit by hospital coders and on provider documentation. The nonspecific UTI diagnosis code 599.0 was commonly used and thus determination had to be made whether the case represented cystitis or pyelonephritis as treatment choice and duration were dependent on the specific urinary tract syndrome being treated. Based on proposed classification systems in the literature [24], [25], provider determined UTI diagnoses with the nonspecific 599.0 code were classified as cystitis if there was no documentation of fever, flank pain or costovertebral angle tenderness, if the note specifically stated that no evidence for pyelonephritis was present, or if the presence of fever, flank pain or costovertebral angle tenderness was attributed to another condition. If fever or flank pain was present without documentation of an alternative diagnosis for these signs or symptoms, then the visit was classified as pyelonephritis.

Patients’ medical records were reviewed for 8 weeks following the ED visit to evaluate for treatment failure and for adverse events related to treatment. Treatment failure was defined as a change in the initially prescribed UTI treatment regimen within 2 weeks of prescription due to clinical failure, isolation of a resistant organism, or adverse effects. Primary adverse events included allergic reactions, gastrointestinal side effects, and vaginal yeast infections occurring within 8 weeks of treatment and deemed to be related to the antibiotic prescribed for UTI. Other adverse events included return visits to the ED or other hospital sites for persistence of UTI symptoms or for additional treatment for alternative diagnoses related to UTI symptoms, missed STI diagnoses, or for treatment of a recurrent UTI (separate from initial UTI) occurring within 8 weeks of the initial ED visit. For diagnostic accuracy, patients were classified as having definite/probable, possible, unlikely, and rejected diagnoses of UTI based on defined criteria (Table S1). Questionable classifications were resolved by a second reviewer.

### Electronic order set intervention (period 1)

In December 2010, recommendations for management of uncomplicated UTIs, based on the anticipated updated IDSA and European Society for Microbiology and Infectious Diseases guidelines [14] (subsequently published in March 2011), were reviewed with three key ED personnel. Electronic versions of the IDSA and European Society for Microbiology and Infectious Diseases guidelines were sent to the three key ED personnel after they were published. From our institution’s traditional antibiogram, which reports organism susceptibility data from the first isolate per patient per year from any anatomic site and any hospital site (both inpatient and outpatient), the overall rate of *Escherichia coli* resistance to TMP-SMX was 19%, less than the 20% threshold for including TMP-SMX as a first line therapy for uncomplicated cystitis [14]. Therefore, 3 days of TMP-SMX or 5 days of nitrofurantoin was recommended as first line therapy for uncomplicated cystitis. The institution at which this study occurred serves an indigent population. Fosfomycin was not included as a primary treatment option due to its expense and due to the fact that *Escherichia coli* susceptibility rates to nitrofurantoin at our institution are high. A 3 day course of ciprofloxacin was reserved for patients in whom both TMP-SMX and nitrofurantoin were contraindicated. Contraindications to TMP-SMX or nitrofurantoin included significant allergies or intolerances or a urine culture within the last year with an organism that was resistant to both TMP-SMX and nitrofurantoin.

The recommended treatment regimen for pyelonephritis was ciprofloxacin for 7 days or TMP-SMX for 10 – 14 days. There was no formal recommendation for an initial IV dose; however a recommendation for a urine culture in cases of pyelonephritis was included. A UTI electronic order set including these recommendations was created and made available for use on December 30, 2010 (Figure S1 provides a screen shot of the electronic order set). ED personnel were informed of the order set by the ED department chair and the physician responsible for medical informatics for the ED.

The ED had a pre-existing policy in place for providing small financial incentives to faculty providers based on their compliance with several quality indicators. Although not part of our stewardship initiative, in April 2011, three months after the implementation of the order set and after collection of the first two period 1 data points, ED personnel decided to include use of the UTI order set as one of their quality indicators. Financial incentive checks related to use of the order set were first received in August 2011.

### Audit and feedback intervention (period 2)

From November 14, 2011 until January 16, 2012 we performed an audit and feedback intervention, in which charts of women meeting study eligibility as described previously were reviewed. A pharmacist (AS) reviewed the cases and discussed them with the ID physician (MH). This required approximately 1.5–3 hours of pharmacist time and 30 minutes of physician time per day (Monday through Friday). Feedback for individual patient visits was given to providers via staff messages in the EMR within 5–7 days of the ED visit if the recommended UTI medication choice or duration of therapy was not used, if urine cultures were not sent in cases of suspected pyelonephritis, or if the UTI diagnosis was determined to be unlikely or rejected by defined UTI study criteria. An educational lecture about UTIs was given to the ED physicians on January 18, 2012. Information presented included a detailed review of the IDSA and European Society for Microbiology and Infectious Diseases treatment guidelines, definitions for various UTI syndromes including uncomplicated vs. complicated UTIs and asymptomatic bacteriuria, data from the baseline and period 1 time periods, and examples of cases that we defined as unlikely or rejected.,

### Outcome measures

The primary outcome was overall adherence to guidelines for management of uncomplicated UTI, both to medication choice and duration of therapy. Secondary outcomes included total and unnecessary days of antibiotic therapy for UTI, use of fluoroquinolones for uncomplicated cystitis, treatment failure, adverse events, and diagnostic accuracy.

### Statistical analysis

Data were analyzed with the use of STATA 11 (StataCorp). We compared the baseline with period 1 and period 1 with period 2 using unpaired Student *t*- and Kruskal-Wallis tests for normally- and non-normally distributed data, respectively. The Pearson Chi-square test and Fisher’s exact tests were used for categorical data as indicated.

## Results

### Characteristics of study subjects

There were no significant differences in patient characteristics between baseline, period 1, and period 2 (Table 1). Overall, 66% to 74% of visits were classified as cystitis. During the 3 time periods, patients were assessed by 32, 37, and 40 different providers, respectively. During the 2 months of audit and feedback, 127 patient visits meeting inclusion criteria were reviewed. Sixty seven recommendations were made for 51 patient visits (40%). Types of recommendations given included: 16 (24%) for alternate medication choice, 11 (16%) for alternate duration of therapy, 7 (11%) for urine culture for pyelonephritis, and 33 (49%) for diagnosis unlikely/rejected.

**Table 1:** Patient characteristics by study time period.

		Baseline	Period 1	Period 2	*P* value	
Characteristic		*n* = 200	*n* = 200	*n* = 200	Baseline– period 1	Period 1- period 2
Age, median, (IQR 25–75)		30 [22–43]	27 [21–43]	30 [ 22–42]	.66	.75
Age > 50		25 (12.5)	28 (14.0)	23 (11.5)	.66	.45
Ethnicity	Black	69 (34.5)	74 (38.0)	57 (28.5)	.60	.07
	Hispanic/Latino	18 (9.0)	27 (13.9)	24 (12.0)	.15	.65
	White	83 (41.5)	83 (42.6)	57 (28.5)	1.00	.006
Diabetes		12 (6.0)	13 (6.5)	10 (5.0)	.84	.52
Renal conditions^a^		21 (10.5)	23 (11.5)	17 (8.5)	.75	.32
History of STI^b^		47 (23.5)	52 (26.0)	62 (31.0)	.56	.27
Post-menopausal^c^		26 (13.0)	25 (12.5)	21(10.5)	.40	.27
Hospitalization within the past 12 months		30 (15.0)	22 (11.0)	20 (10.0)	.31	.57
Antibiotic use within the past 12 months	Any	97 (48.5)	103 (51.5)	100 (50.0)	.55	.76
	Trimethoprim-sulfamethoxazole	28 (14.0)	35 (17.5)	39 (19.5)	.34	.61
	Ciprofloxacin	34 (17.0)	29 (14.5)	20 (10.0)	.49	.17
	Nitrofurantoin	8 (4.0)	16 (8.0)	15 (7.5)	.09	.85
Diagnosis	Cystitis	133 (66.5)	131 (65.5)	148 (74.0)	.83	.06
	Pyelonephritis	67 (33.5)	69 (34.5)	52 (26.0)	.83	.06

Data are no. or proportion (%) of patients, unless otherwise indicated.

a Renal conditions include history of kidney stones, recent acute or chronic kidney disease, urinary incontinence with or without previous surgery for this condition.

b STI, sexually transmitted infection.

c Documented post-menopausal status or age > 50 if status not documented.

### Adherence to guidelines

**Figure 1:**
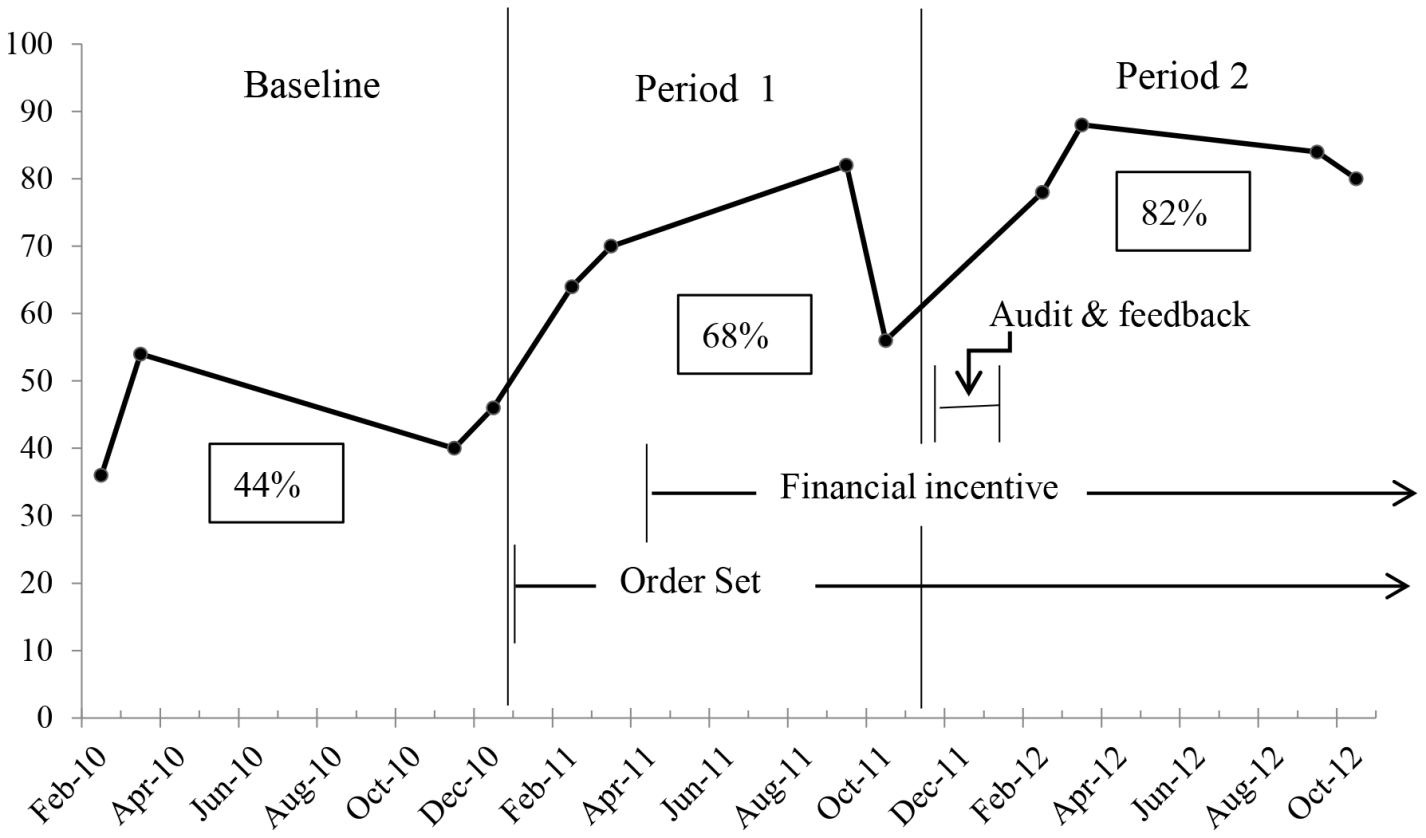
Percentage of cases adherent to UTI guidelines at baseline and during the tiered stewardship intervention. The percentages in the boxes are the average of the 4 time points during each period.

Overall adherence to UTI treatment recommendations with regard to both antibiotic choice and duration of therapy at baseline and during the intervention periods is shown in Figure 1. In comparison to baseline, after implementation of the electronic order set (period 1) overall adherence to recommended UTI treatment regimens increased from 44% to 68% (*P*<.001), adherence to the recommended antibiotic choice increased from 70% to 89% (*P*<.001), and adherence to the recommended duration of treatment improved from 62% to 75% (*P* = .005). There was no difference in adherence during period 1 before versus after the financial incentive was added for use of the order set (67% versus 69%; *P* = .88).

In comparison to period 1, after audit and feedback overall adherence to treatment guidelines increased further from 68 % to 82% (*P* = .015), that was attributable to increased adherence to the recommended duration of treatment from 75% to 88% (*P* = .017). Adherence to recommended antibiotic choice was similar in periods 1 and 2 (89% and 90%, respectively; *P* = .74).

In comparison to the baseline period, there was a significant increase in the percentage of urine cultures sent for cases of suspected pyelonephritis during the combined intervention periods (25/67, 37% versus 70/121, 58%, respectively; *P* = .007). There was no increase in the percentage of urine cultures sent for cases of suspected cystitis during the intervention periods (36/133, 27% versus 71/278, 26%, respectively; *P* = .74).

### Antimicrobial use

In comparison to baseline, after implementation of the electronic order set (period 1), unnecessary UTI days of therapy for the 200 patients evaluated in each period decreased from 250 unnecessary days to 119 unnecessary days (*P*<.001), prescription of fluoroquinolones for uncomplicated cystitis decreased from 44% to 14% (*P*<.001), and the average duration of TMP-SMX or ciprofloxacin therapy for cystitis decreased from 5.6 days at baseline to 3.9 days (*P<*.001) (Table 2).

**Table 2:** Antibiotic use, treatment failures, and adverse events for study patients at baseline and during intervention Period 1 and Period 2.

				*P* value
**Outcome**	BaselineN = 200	Period 1N = 200	Period 2N = 200	Baseline-Period 1	Period 1-Period 2
Mean duration of therapy for cystitis^a^ (days)	5.6	3.9	3.6	<.001	.17
Percentage of fluoroquinolone regimens for cystitis	44.4	14.5	12.9	<.001	.70
Unnecessary antibiotic days of therapy, total	250	119	52	<.001	<.001
Treatment failure^b^	22 (11.0)	14 (7.0)	17 (8.5)	.16	.58
Primary adverse events^c^	12 (6)	17 (8.5)	19 (9.5)	.66	.59
Other adverse events^d^	49 (24.5)	54 (27)	47 (23.5)	.54	.52

a Including only trimethoprim-sulfamethoxazole and ciprofloxacin containing regimens.

b A change in the initially prescribed UTI treatment regimen within 2 weeks of prescription due to clinical failure, isolation of a resistant organism, or adverse effects.

c Including allergic reactions, gastrointestinal side effects, and vaginal yeast infections occurring within 8 weeks of treatment and felt to be related to the antibiotic prescribed for UTI.

d Including return visits to the ED or other hospital sites for persistence of UTI symptoms or for additional treatment for alternative diagnoses related to UTI symptoms, missed STI diagnoses, or for treatment of a recurrent UTI (separate from initial UTI) occurring within 8 weeks of the initial ED visit.

In comparison to period 1, after audit and feedback, unnecessary UTI days of therapy for the 200 patients evaluated decreased significantly further to 52 unnecessary days (*P*<.001), prescription of fluoroquinolones for uncomplicated cystitis remained relatively stable at 13% (*P* = .70) and the average duration of TMP-SMX or ciprofloxacin therapy for cystitis decreased from 3.9 to 3.6 days (*P* = .17) (Table 2).

### Treatment failures and adverse outcomes

Treatment failure occurred in 11%, 7%, and 8% of patients reviewed during the baseline period, period 1, and period 2, respectively (*P* ≥.16 for each comparison). A primary adverse event of either allergic reaction, gastrointestinal side effect, or vaginal yeast infection occurred in 6%, 8%, and 10% of patients reviewed during the baseline period, period 1, and period 2, respectively (*P* ≥.59 for each comparison). Other adverse events occurred in 24%, 27%, and 24% of patients reviewed during the baseline period, period 1, and period 2, respectively (*P*≥.52 for each comparison) (Table 2). Although not included as an outcome, antimicrobial resistance rates for *Escherichia coli* obtained from the institution’s traditional antibiogram over the time period of the study 2010, 2011 and 2012 to TMP-SMX were 19%, 20%, and 21%, to nitrofurantoin were 5%,7%, and 9% and for ciprofloxacin were 16%, 16%, and 16% respectively.

### Diagnostic accuracy

**Figure 2:**
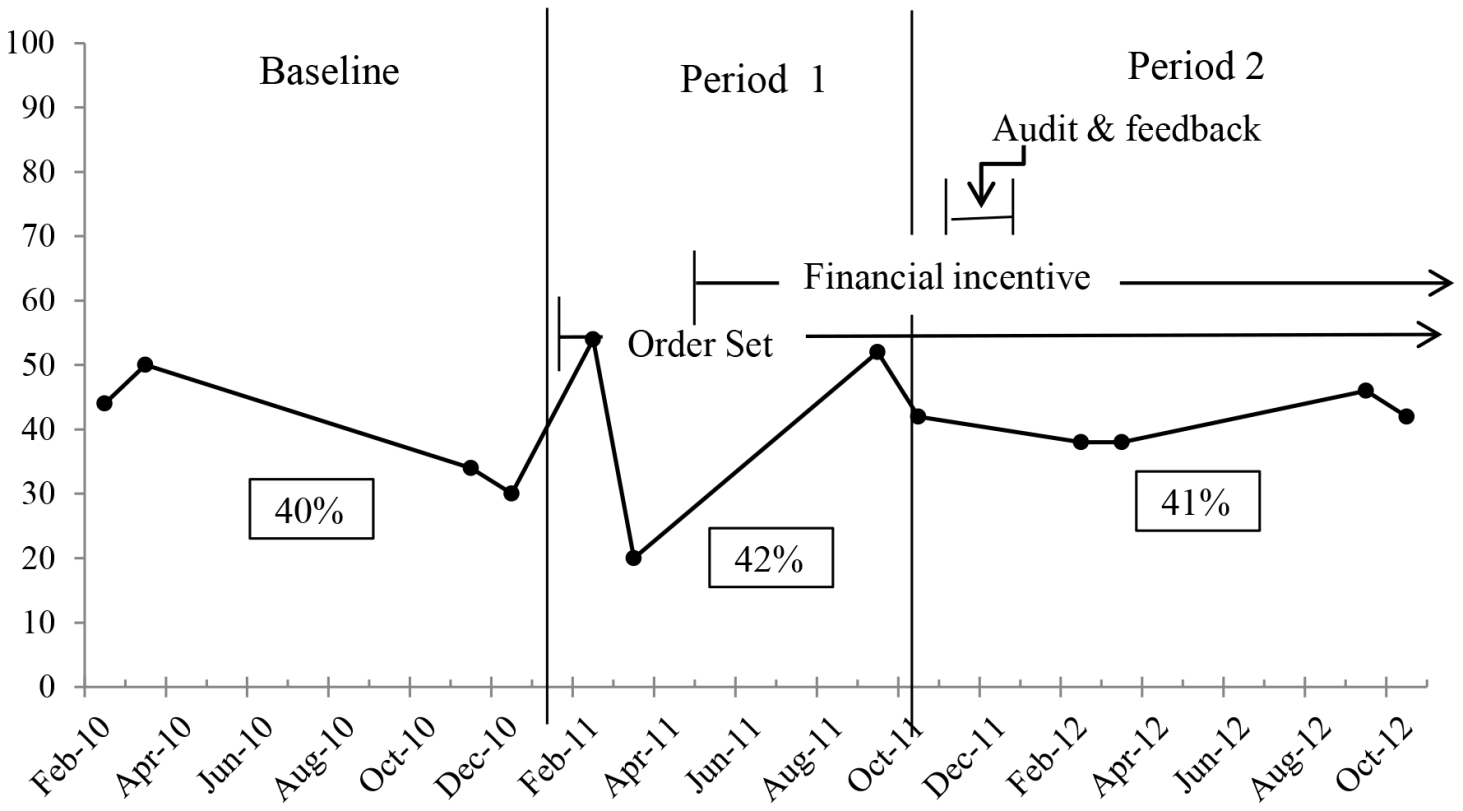
Percentage of cases deemed unlikely or rejected at baseline and during the tiered stewardship intervention. The percentages in the boxes are the average of the 4 time points during each period.

Forty percent, 42%, and 41% of cases diagnosed as UTI by clinicians were classified as unlikely or rejected cases of UTI during the baseline period, period 1, and period 2, respectively (*P* ≥.37 for each comparison) (Figure 2). Examples of cases classified as unlikely or rejected are described in Table 3. Most unlikely or rejected cases had more than one reason to be classified as such. For 245 total cases deemed unlikely or rejected, the reasons they were deemed unlikely or rejected included: 126 (51%) in which patients reported having no urinary symptoms, 50 (20%) had a definite genital tract infection; 59 (24%) had vaginal symptoms attributed to either a genital tract infection for which testing was not done or a non-infectious genital tract syndrome, 47 (19%) had no documentation of urinary symptoms, 10 (4%) had a definite non-genital tract infection to explain the symptoms, 69 (28%) had a probable non-genital tract alternative diagnosis. Urine cultures were done in 40 (16%) cases and all were negative. Negative urinalyses with no urine culture performed occurred in 19 (8%) cases. No improvement despite adequate therapy was seen in 3 (1%) patients.

**Table 3:** Examples of cases diagnosed as urinary tract infection by providers, but classified as unlikely or rejected based on medical record review.

**Description**	**Classification**	**Reason for classification**
20 year old woman diagnosed with genital herpes infection one day ago presented with continued dysuria and difficulty urinating despite acyclovir treatment. Tender genital ulcers on exam. Urinalysis (UA) with 30–100 white blood cells (WBC) per high power field and nitrite negative. Diagnosed with urinary retention due to primary herpes infection and UTI and prescribed 7 days of ciprofloxacin. Urine culture negative.	Rejected	Definite alternative diagnosis and negative urine culture
19 year old woman presented with suicidal ideation. No urinary symptoms, but UA ordered due to mild leukocytosis (13,500 WBC/mL) and because Psychiatry recommended ruling out infection.* UA with negative nitrite, positive leukocyte esterase, 30–100 WBC and 10–30 squamous epithelial cells per high power field. Diagnosed with depression with suicidal ideation and UTI and prescribed 3 days of trimethoprim-sulfamethoxazole. No urine culture sent. The patient was diagnosed with chlamydia 5 months later.	Unlikely	No urinary symptoms
21 year old with motor vehicle accident 2 months ago presented with lower back pain exacerbated by movement and right foot numbness. No urinary symptoms or fever. UA with negative blood and nitrite, 10–30 WBC and 10–30 squamous epithelial cells per high power field. Diagnosed with acute lumbar strain and UTI and prescribed 7 days of nitrofurantoin. No urine culture sent.	Unlikely	Probable alternative diagnosis and no significanturinary symptoms
65 year old post-menopausal woman presented with heavy vaginal bleeding, but no urinary symptoms or fever. On exam a mass was noted protruding from the cervical os. Clean catch UA with large blood, large leukocyte esterase, positive nitrite, 10–30 WBC and 2–5 squamous epithelial cells per high power field. Diagnosed with vaginal bleeding (suspected cervical cancer) and UTI based on abnormal UA and prescribed 5 days of nitrofurantoin. Straight catheterization urine culture negative.	Rejected	Vaginal symptoms and definitive alternative diagnosis. Urine culture negative.
33 year old woman presented with epigastric burning pain, nausea and vomiting. No urinary symptoms, fever, or flank pain. Mild epigastric tenderness on exam. UA with negative blood, positive nitrite, small leukocyte esterase, 2–5 WBC and 30–100 squamous epithelial cells per high-power field. Diagnosed with dehydration and UTI and prescribed 7 days ciprofloxacin. No urine culture sent.	Unlikely	Probable alternative diagnosis (GI) and no urinary symptoms
33 year old woman presented with fever, cough, headache, myalgias, back pain, and sick contacts with similar illness. No urinary symptoms. On exam, temperature was 38.5 °C. There was neither abdominal tenderness nor costovertebral angle tenderness. UA not done, but the provider listed abnormal findings from a UA from 6 months earlier with pyuria (10–30 WBC per high power field). Diagnosed with viral syndrome and UTI and prescribed trimethoprim-sulfamethoxazole for 3 days.	Unlikely	Probable alternative diagnosis (influenza-like illness during influenza season)

* Interviews with Emergency Department providers suggested that Psychiatry personnel recommended sending a UA to rule out “infection” in anyone presenting with psychiatric illness.

## Discussion

We demonstrated that a stewardship intervention that included implementation of an electronic order set followed by a period of audit and feedback was associated with a sustained improvement in adherence to uncomplicated UTI guidelines. These findings have important implications for several reasons. First, the improvement in adherence to guidelines was achieved through a simple intervention that required minimal resources. Because the EPIC® EMR system is widely used, similar order sets could be instituted in many other U.S. healthcare facilities. Second, because UTI is one of the most common indications for prescription of antimicrobials, improving management of this condition should be a focus of stewardship programs. Our intervention reduced both unnecessary antibiotic days of therapy for UTI and fluoroquinolone use for cystitis. Reducing overuse of fluoroquinolones is an important goal due to their propensity to cause adverse ecological effects and due to rising rates of resistance to these agents [26]. Finally, the improvement in adherence to UTI guidelines was achieved without any apparent increase in treatment failures or adverse events.

Despite the increase in adherence to UTI treatment guidelines, it is notable that there was no improvement in UTI diagnostic accuracy during the intervention. For 40% to 42% of cases diagnosed as UTI by clinicians, the diagnosis was deemed unlikely or rejected, consistent with previous studies [20]–[23]. The lack of improvement in diagnostic accuracy with the audit and feedback intervention may have been in part due to its relatively short duration (2 months) and that the feedback was not given in real time. However, it is likely that diagnostic accuracy is more difficult to improve as compared to choosing the appropriate medication and duration of therapy.

One strategy to optimize diagnostic accuracy might be to improve use and interpretation of the urinalysis. In our ED, the protocol followed by the triage nurses requires a urinalysis to be performed in any woman presenting with abdominal pain, urinary tract symptoms, genital tract symptoms, or fever. Based upon clinical documentation and discussion with ED providers during audit and feedback, treatment of UTI cases classified as unlikely or rejected was often driven by the finding of a “dirty urine” (i.e., abnormal urinalysis). Patients who did not have symptoms suggestive of a UTI or who had alternative explanations for their symptoms (e.g. genital tract infections) were often treated for a UTI solely on the basis of an abnormal urinalysis.

Our findings add to previous reports of interventions that resulted in improved adherence to UTI guidelines in ambulatory clinic settings [27]–[30]. The two studies performed in the United States were published over 10 years ago prior to the IDSA UTI guidelines [27], [28]. These studies included select groups of patients with a high likelihood of cystitis, demonstrated improvement primarily through nurse driven protocols either over the telephone or at an office visit, focused on reducing lab testing for uncomplicated cystitis, and alluded to but did not fully assess the problem of overdiagnosis of UTI. Although Saint et al. [27] evaluated a large number of patients, the data was reviewed on a population level without review of individual patient information. These studies also involved significant educational components with multiple meetings and ancillary tools. In contrast to previous studies, our study describes a more “real world” view of the management of uncomplicated UTIs (both cystitis and pyelonephritis) that was effective in changing physician behavior. We did not focus on reducing lab testing for cystitis but instead on improving testing for pyelonephritis and we assessed the significant problem of overdiagnosis of UTI. We also utilized the electronic medical record to implement our intervention with few additional ancillary efforts. Our study also was performed in an ED setting as compared to ambulatory clinic settings in the previous studies. Finally, in contrast to our findings, Flottorp et al. [29] found that a multi-faceted intervention that included computer based decision support had little effect in improving adherence to UTI treatment guidelines.

Our study has several limitations. First, the study was conducted at a single institution and the intervention was specific to the department workflows (frequent use of electronic order sets) and patient population (urban population with high rates of sexually transmitted infections) of that institution. Other approaches may be better suited to different institutions or departments. Second, it is not clear if the small financial incentive for use of order sets contributed significantly to the success of the intervention. However, within period 1 there was no difference in adherence to the guidelines before versus after the financial incentive was implemented. Third, urine cultures were not routinely performed as part of the evaluation for suspected UTI. Thus, determination regarding diagnostic accuracy was often dependent on urinalysis results and documentation of signs and symptoms. Fourth, the criteria we used to determine diagnostic accuracy have not yet been validated. Fifth, we did not provide definitions for uncomplicated vs. complicated UTIs in the electronic order set and did not evaluate ED visits for patients with complicated UTIs to determine if they were treated inappropriately as uncomplicated UTIs. Finally, the use of ICD9 codes could overestimate or underestimate the diagnosis of UTI since it is dependent on the hospital coder; however no changes in the coders or how they apply the codes were made during the study period and thus although the absolute numbers may be over or under-estimated, the same rate of over and under-estimation would be assumed to occur over time and thus the trends seen would most likely not be related to changes in over or under-estimation

In summary, an antimicrobial stewardship initiative for uncomplicated UTI was associated with a significant and sustained improvement in adherence to treatment guidelines, a decrease in unnecessary antibiotic therapy for UTIs, and a reduction in the use of fluoroquinolone containing regimens for uncomplicated cystitis. There was no increase in UTI treatment failures or adverse events. Future studies are needed both to determine the effect of this intervention on rates of antimicrobial resistance in uropathogens and to identify effective strategies to reduce the frequency of prescription of UTI therapy for patients who are unlikely to have UTIs.

## Supporting Information

Figure S1 **Screen shot of the electronic order set.**
(TIF)

Table S1 **Criteria for Study Defined UTI Diagnostic Classifications.**
(DOCX)
