# Supplementary material for: Effect of a Stewardship Intervention on Adherence to Uncomplicated Cystitis and Pyelonephritis Guidelines in an Emergency Department Setting
Source: PLoS One. 2014 Feb 3;9(2):e87899. doi: 10.1371/journal.pone.0087899 (PMC3912125; doi:10.1371/journal.pone.0087899)
Supplement: Table S1 — Criteria for Study Defined UTI Diagnostic Classifications. (DOCX) [file pone.0087899.s002.docx]

**Table S1**. Criteria for Study Defined UTI Diagnostic Classifications

| **Cystitis** |  |
| --- | --- |
|  |  |
| Definite/probable | - Presence of dysuria or other significant urinary symptoms |
| All 4 criteria required | - Absence of significant vaginal signs/symptoms^a^ |
|  | - Positive urinalysis^b^ or positive urine culture |
|  | - Absence of an alternative diagnosis |
|  |  |
| Possible | - Not meeting criteria for other categories |
|  |  |
| Unlikely | - Absence of significant urinary symptoms |
| Any of the 4 required | - Presence of significant vaginal signs/symptoms^a^ |
|  | - Negative urinalysis^c^ |
|  | - Probable alternative diagnosis |
|  |  |
| Rejected | - Definitive alternative diagnosis |
| Any of the 3 criteria required | - Negative urine culture in the absence of antibiotics within the last week |
|  | - No resolution/improvement in signs/symptoms despite adequate UTI treatment. |
|  |  |
| **Pyelonephritis** |  |
|  |  |
| Definite/probable | - Temperature > 37.8 or reported fever/chills or leukocytosis |
| All 5 criteria required | - Either flank pain or significant urinary symptoms |
|  | - Absence of significant vaginal signs/symptoms^a^ |
|  | - Positive urinalysis^b^ |
|  | - Absence of alternative diagnosis |
|  |  |
| Possible | - Not meeting criteria for other categories |
|  |  |
| Unlikely  Any of the 4 criteria required | - Absence of temperature > 37.8, reported fever/chills, leukocytosis, or flank pain |
|  | - Presence of significant vaginal signs/symptoms^a^ |
|  | - Negative urinalysis^c^ |
|  | - Probable alternative diagnosis |
|  |  |
| Rejected | - Definitive alternative diagnosis |
| Any of the 3 criteria required | - Urine culture negative in the absence of antibiotics within the last week OR |
|  | - No resolution/improvement of symptoms/signs in follow up despite adequate UTI treatment |

^a^ in the absence of a positive urine culture

^b^a positive urinalysis = presence of at least one of the following; positive nitrite, positive leukocyte esterase or > 5 wbc/hpf

^c^a negative urinalysis = negative nitrite, negative leukocyte esterase and if done < 5 wbc/hpf

Consider leukocyte esterase=trace as negative if wbc < 5 wbc/hpf
